# Experimental Methods for Studying Cellular Heme Signaling

**DOI:** 10.3390/cells7060047

**Published:** 2018-05-24

**Authors:** Jonathan M. Comer, Li Zhang

**Affiliations:** Department of Biological Sciences, School of Natural Sciences and Mathematics, The University of Texas at Dallas, Richardson, TX 75080, USA; jonathan.comer@utdallas.edu

**Keywords:** heme, transcriptional regulation, signaling, heme detection, heme-protein interactions

## Abstract

The study of heme is important to our understanding of cellular bioenergetics, especially in cancer cells. The function of heme as a prosthetic group in proteins such as cytochromes is now well-documented. Less is known, however, about its role as a regulator of metabolic and energetic pathways. This is due in part to some inherent difficulties in studying heme. Due to its slightly amphiphilic nature, heme is a “sticky” molecule which can easily bind non-specifically to proteins. In addition, heme tends to dimerize, oxidize, and aggregate in purely aqueous solutions; therefore, there are constraints on buffer composition and concentrations. Despite these difficulties, our knowledge of heme’s regulatory role continues to grow. This review sums up the latest methods used to study reversible heme binding. Heme-regulated proteins will also be reviewed, as well as a system for imaging the cellular localization of heme.

## 1. Introduction

One of the key characteristics of cancer cells is their need for nutrients to supply energy and metabolic building blocks. Mitochondrial respiration and oxygen consumption play a crucial role in cancer cell energetics and metabolism [1,2,3,4,5]. Heme is of central importance to oxidative phosphorylation, oxygen consumption, and metabolic regulation. Heme is a small biomolecule produced and used by living organisms from bacteria to humans [6,7]. It consists of an organic porphyrin ring that coordinates an iron ion, Fe(II) or Fe(III), in its center [8]. Heme can be in an oxidized or reduced state and is used extensively by organisms in a variety of redox reactions. As a cofactor in proteins of the electron transport chain, heme is crucial for cellular respiration and energy production. Heme is also the oxygen binding cofactor in globins such as hemoglobin and myoglobin, allowing organisms to distribute oxygen. Heme also acts as an important signaling molecule, bound by a variety of proteins which control many genetic pathways including oxidative stress response and carbon metabolism [6,7]. In cytochromes, such as P450s and cytochrome c, heme is used in the synthesis and degradation pathways of sterols, lipids, and neurotransmitters [9]. Various epidemiological and molecular studies suggest links between heme and several cancers, including colorectal, pancreatic, lung, and endometrial cancers, as well as other diseases such as type-2 diabetes and coronary heart disease [10]. In order to better understand these diseases and develop better treatments, it is important to understand the role of heme. Much is known about the role of heme as a functional prosthetic group, but less is known about its role as a signaling molecule. This review aims to facilitate the study of heme signaling by providing insight into the common methods, problems, and solutions in studying heme signaling.

## 2. Body

### 2.1. Heme Regulatory Pathways

**Figure 1:**
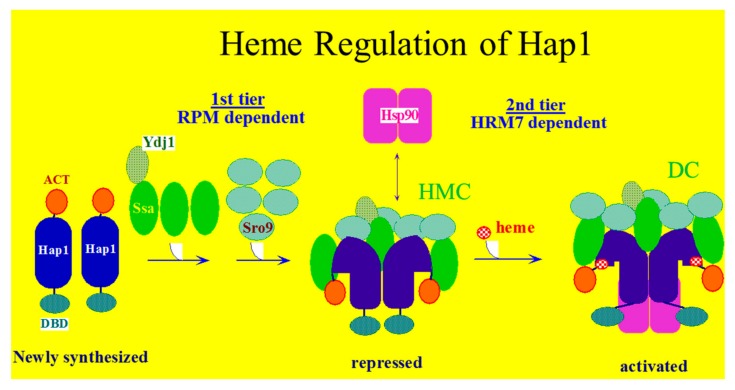
Hap1 repression modules (RPMs) promote Hap1 association with Ydj1, Ssa (Hsp70), and Sro9 to form an inactive High Molecular Weight Complex (HMC). Upon heme binding to Heme Responsive Motif 7 (HRM7), Hsp90 is stably bound and the HMC is disrupted, producing the active dimeric complex (DC). In the new complex, the Hap1 acidic activation domains (ACT) are activated and DNA binding is promoted via the DNA binding domains (DBD).

Several signaling pathways have been linked to heme regulation via reversible heme-protein binding. Heme-activating protein, Hap1, is one of the earliest regulatory heme-binding proteins studied and plays a central role in *S. cerivisiae*’s response to oxygen levels [6,11]. Hap1, a transcription factor, accomplishes this by direct interaction and sensing of heme concentrations [7]. Hap1 contains several domains allowing it to perform varying levels of gene activation in response to heme [11]. Hap1 contains a DNA binding domain and transcriptional activation domain as well as a coiled-coil domain and a domain instrumental in repressing Hap1’s transcriptional activity. Hap1 forms an inactive high-molecular-weight complex with chaperones Hsp70, Sro9, Ydj1. Upon binding to heme, the complex activates by binding to Hsp90 [7]. It has been found that heme directly regulates Hap1 DNA binding, as well as Hap1’s transcriptional activating domain through two distinct sets of Heme-Responsive-Motifs (HRMs). Six HRMs near the DNA binding domain regulate binding of Hap1 to DNA, and appear to have a small impact on Hap1 activity. Most of Hap1’s activity is mediated through HRM7 near the acidic transcription activation domain and repression modules. This HRM regulates the coiled-coil-based dimerization of Hap1 and recruitment of Hsp90 to the complex. This two-level activation and de-repression of Hap1 (see Figure 1) provides a fine-tuned response to a range of heme concentrations [7,12].

Mammalian reticulocytes (red blood cell precursors) produce a large amount of hemoglobin as they develop into mature erythrocytes. This requires synchronized production of both globin chains and heme to avoid toxic accumulation of unassembled globin chains [7,13]. This synchronization is accomplished by heme regulation of global protein synthesis via the heme-regulated eIF2α kinase (HRI). Under heme deficient conditions, translation of globin chains is reduced by HRI kinase to match the lower levels of heme. Under these conditions, HRI kinase becomes active and phosphorylates the translational initiation factor eIF2alpha, preventing it from being recycled. This effectively inhibits translation and thus synthesis of globin chains and other proteins [13,14]. Inactive HRI is complexed with chaperones Hsp90 and Hsc70. Upon activation by heme, HRI gains eIF2alpha kinase activity and autokinase activity and is no longer associated with the chaperones [7,13]. Regulatory heme binding is thought to occur at an HRM in the kinase insertion sequence (KI) domain. This domain and HRM are situated between two halves of the catalytic domain. HRI activation is thought to occur by bringing together the two halves of the catalytic domain. There is another regulatory site in the N-terminal domain which imparts nitric oxide and carbon monoxide sensitivity to HRI. This site binds heme stably and does not contain the CP motif typical of reversible heme binding sites. There is an HRM in the C-terminal domain, but this domain does not bind heme [7,15,16].

Other mammalian cells need to respond to heme concentrations as well. Bach1 is a transcriptional repressor that is regulated directly by heme. Bach1 regulates genes related to oxidative stress response, globins, heme oxygenase-1, as well as erythroid specific ALAS [17,18]. Like Hap1, Bach1 is regulated through a multifaceted mechanism involving heterodimers, DNA binding, and subcellular localization. Bach1 is a basic leucine zipper protein and has two domains: the protein interaction domain (BTB/POZ) and the domain which regulates DNA binding (bZIP). There are six HRMs, two in the vicinity of the BTB domain, and four near the bZIP domain. One method of heme regulation of Bach1 appears to be through nuclear export via heme-activated nuclear export signals involving three of the HRMs. Another mechanism is through heme inhibition of DNA binding of the Bach1-MafK heterodimer. Bach1 forms heterodimers with the Maf-related oncoprotein family of proteins. These dimers then bind the Maf recognition element (MARE) of genes involved in heme and oxygen metabolism [7,17,18,19,20].

**Figure 2:**
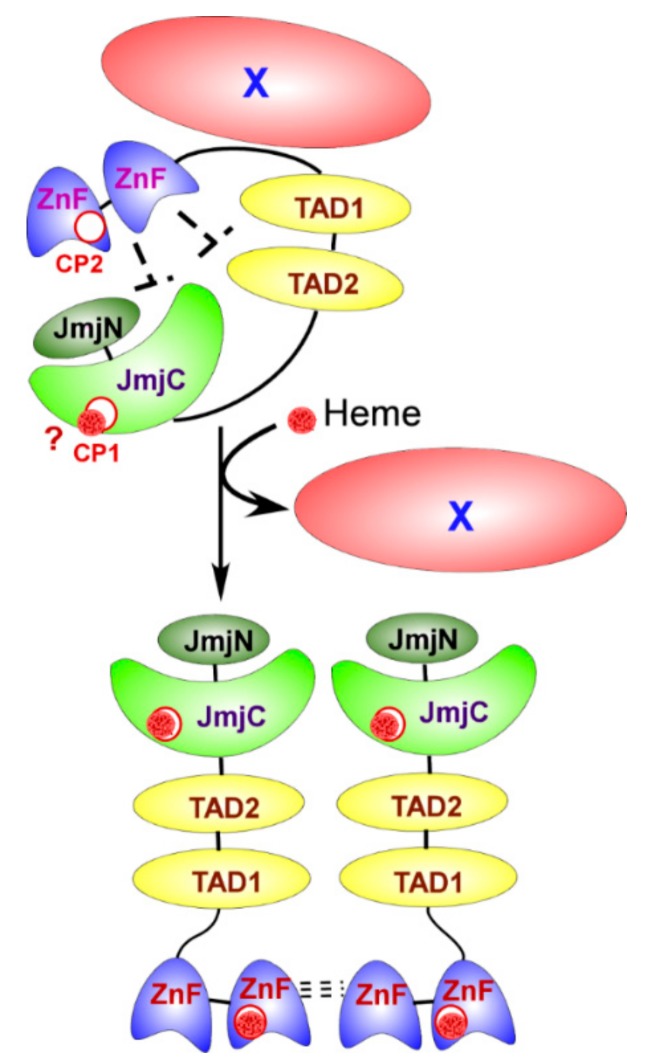
At low heme levels, the ZnF region and other proteins (X) repress Gis1 transcriptional and demethylase activities. At higher heme concentrations, heme binds a second site in the ZnF causing loss of X, oligomerization of Gis1, and conformational changes which fully activate Gis1 demethylase and transcriptional activities. JmjN and JmjC: jumonji domains and demethylase activity. TAD1 and TAD2: transcription activation domains. ZnF: C2H2 type zinc fingers.

Gis1 is a heme-regulated transcriptional regulator and demethylase in *S. cerevisiae*. It is orthologous to the mammalian JMJD2/KDM4 family of demethylases whose dysfunction has been connected with cancer and cardiovascular disease [21,22]. Gis1 binds the Post Diauxic Shift (PDS) element and regulates a large number of genes involved in oxidative stress response and carbon metabolism [23,24,25,26]. Gis1 has been shown to have two heme binding sites, and both its demethylase activity and transcriptional activating activity are activated by heme (see Figure 2) [27]. From these four examples, Hap1, HRI, Bach1, and Gis1, we see that heme is used as a signaling molecule which impacts the expression of many genes and can dramatically alter cellular metabolism. These proteins also display an interesting variety in the mechanisms used to detect heme, providing some insight into the mechanisms to search for in other heme-regulated proteins.

### 2.2. Measuring K_d_

The affinity of a protein or peptide for heme is often expressed as the dissociation constant of the heme-protein complex, K_d_. K_d_ can be determined by measuring the equilibrium concentrations of free and bound components while varying the concentration of one component. A simple example is to monitor the amount of bound heme by spectroscopy while titrating with a heme-binding protein. The experiment can then be visualized by plotting the concentration of bound heme vs. the concentration of protein. K_d_ would be calculated from the concentrations of free protein, free heme, and heme:protein complex. Some concentrations may not be known from the experimental data (such as free protein and free heme), thus various mathematical substitutions are made in the calculation of K_d_. In order to accurately determine the number of heme binding sites and their affinity, many data points must be taken over a wide range of heme:protein ratios with special focus on concentrations near the K_d_ (s). Insufficient or poorly planned data points may lead to incorrect or oversimplified conclusions [28]. Thordarson provides a thorough discussion of choosing data points to accurately measure association constants. It is critical to cover a sufficiently large range of heme:protein concentration ratios in order to ensure accuracy of the results. It is just as crucial to take sufficient data points within that range to distinguish multiple binding sites, non-specific binding, or cooperative binding. The suggested starting setup to study unknown reversible heme binding is to take ~10 data points up to a ratio of 1:1.5 protein:heme, then ~10 data points up to a ratio of 1:50, or at least 1:10 [28]. Finally, appropriate analysis of the resulting data is necessary to achieve accurate results. Given the modern availability and speed of computer software, it is recommended to use non-linear regression and various binding models in order to accurately analyze binding data [28]. Linear regression methods are statistically undependable because the result is heavily weighted towards the most extreme data points. Also, heme sensing proteins often have multiple heme binding sites which are not distinguished by a linear regression model. Some of these standards, however, can be very difficult or impossible to follow given the combined constraints of heme solubility, protein solubility, and instrument limitations. Many studies of heme binding proteins are focused on the biological significance of heme sensing, and therefore only need a good estimate of a protein’s affinity for heme. In such cases, approximate or “overall” affinities have been used in combination with experiments to determine biological significance and rule out non-specific binding [19,29,30].

### 2.3. Chemical Background

Heme (iron protoporphyrin IX), consists of an iron ion chelated by a tetrapyrrole ring. Various functional moieties can be substituted on the outer carbons of the ring, but the most common form is heme B [9]. When oxidized, iron protoporphyrin IX is technically called “hemin”, but is often referred to simply as “heme,” as will be done in this paper. Due to the side chains of the tetrapyrrole ring, heme has hydrophobic and hydrophilic ends. This slight amphiphilic property allows heme to bind in hydrophobic binding pockets of proteins. The pi electrons donated by the four nitrogen atoms are shared in the ring and coordinate with the positively charged iron ion in a primarily planar or slightly bent structure. This leaves two orbitals on either face of the molecule available for binding to proteins or small gas molecules such as oxygen, nitric oxide, and carbon monoxide. Heme exhibits strong absorption in the UV-Vis spectrum due to a Soret band near 400 nm. This absorbance is a result of the Π-Π* transition of the porphyrin ring [8,31]. The energy gap of the Π-Π* transition, and thus the shape of the absorption curve from 300 to 600 nm, is sensitive to the coordination of other molecules, such as amino acid residues or bound gas molecules [9]. Absorbance spectroscopy is therefore a simple and highly sensitive technique to detect interactions between heme and proteins [11,27,29,30,32,33,34,35,36,37,38,39,40,41,42].

### 2.4. UV-Vis Spectrophotometry

Because the heme absorption spectrum is highly sensitive to ligand coordination, absorption spectroscopy is one of the favored assays to detect and study reversible heme binding at a biochemical level. One author studied a variety of reversible heme-binding peptides and has classified the absorbance spectra into four groups. This may have significance in distinguishing the type of binding and function of different proteins, as well as distinguishing signals of different heme-binding proteins in the same mixture (e.g., competition assays) [33,34].

In aqueous solutions, heme has low solubility and favors heme dimers at equilibrium. There is also a time-dependent loss of available heme which may be attributed to formation of heme aggregates or to stabilization of heme dimers by auto-oxidation. When dissolved in the organic solvent dimethyl sulfoxide (DMSO), these problems are solved and heme exists in its monomeric state; however, DMSO may have undesired effects on protein stability or protein-heme interactions [43]. In order to favor monomeric heme in physiological buffers, stock solutions of heme are often prepared in DMSO and then freshly diluted into an aqueous sample to ~2% DMSO [43]. Higher concentrations of DMSO (3:5 DMSO:water) have been used when it was determined that protein stability was not affected [42]. Another technique often used to completely avoid organic solvents is to prepare heme stocks in highly basic aqueous buffers and then mix a fresh diluted working stock [36,43]. In addition, due to the higher solubility of heme at higher pH, many experiments are performed under slightly basic conditions. Our lab has found that in a working solution, heme is soluble to about 500 μM in phosphate buffered saline (PBS) and around 50 μM in Tris pH 8.

The heme absorbance spectrum is also affected by some common buffers and additives used in protein purification; therefore, care must be taken when interpreting the spectra. A base spectrum of heme in buffer alone should always be taken for comparison. Reagents which affect the spectrum should be either removed from the sample or included at constant concentrations in all buffers. Dithiothreitol (DTT) and imidazole change the basic shape of the heme spectrum. DTT shifts the heme peak from 390 towards 385 nm. In the presence of 250 mM imidazole, DTT (~1–40 mM) shifts the peak from 434 to 423 nm. Tris(2-carboxyethyl)phosphine (TCEP) may be preferred as a reducing agent due to its higher stability compared to DTT and β-mercaptoethanol (BME). At millimolar concentrations, imidazole has a distinct influence on the heme spectrum, reducing the iron and shifting the peak to 434 nm. Our lab has found the heme absorbance spectrum often decreases with time, requiring a fresh preparation every half hour or so. Imidazole appears to prevent this problem, presumably by reducing heme and preventing its dimerization or aggregation [43]. Another use of imidazole is as a weak competitor for heme interaction, used to approximate the affinity of the protein for heme [27].

Finally, the His6 tag, often used for protein purification, binds heme weakly. Experiments in our lab with purified heme-binding proteins showed a noticeable broad peak around 410 nm when the His6 tag was left intact. Upon cleavage of the His6 tag with thrombin and removal of the cleaved tag via Ni^2+^ affinity purification, the heme absorbance spectrum shifted to around 400 nm, indicating heme binding to the protein rather than the tag. Addition of imidazole at concentrations below ~10 mM did not shift the peak from 410 to 434 nm (the peak for heme and imidazole alone), suggesting that heme binding to the His6 tag is distinguishable from interaction with imidazole. At higher concentrations of imidazole (>20 mM), however, the heme binding proteins did shift the heme absorbance spectrum but the presence or absence of His6 no longer had an effect, indicating the weakness of the His6-heme interaction.

### 2.5. Fluorescence

Heme does not fluoresce, but will quench fluorescence when bound near a fluorescent molecule [44]. If the protein of interest has fluorescent properties (such as a fluorescent tryptophan residue), the quenching effect of bound heme can be used to measure the concentration of heme-protein complex. Like the spectrophotometric absorption method, the K_d_ value of heme binding can be determined via heme titration [42].

### 2.6. Equilibrium Dialysis

Equilibrium dialysis simply involves dialyzing a protein sample against buffer containing a known concentration of heme. After sufficient time has passed for heme to equilibrate across the membrane, the concentration of heme and protein in the sample can be measured. Several separate setups at different concentrations of heme would provide sufficient data to calculate K_d_. The benefits of this method are that it is simple and straightforward, and it is performed at equilibrium, allowing the measurement of reversible interactions. The drawbacks to this method are that only a single data point is generated per setup and a large amount of sample may be used. Also, dialysis is a slow process during which proteins may lose activity or heme may be oxidized. A similar method has been used to qualitatively compare affinities of several proteins for heme, but in this case, time-dependent loss of heme from a protein sample was measured [37]. Such a method does not distinguish dissociation kinetics from affinity, but provides a useful comparison.

### 2.7. Circular Dichroism

Circular dichroism (CD) detects a sample’s chirality by measuring its absorption of circularly polarized light. Just as with other forms of spectroscopy, circular dichroism can be measured over a range of wavelengths to study different chemical attributes. For example, overall protein structure can be detected by CD below 300 nm, where alpha helices, beta strands, and beta turns give unique signatures that combine to form a “fingerprint” of the protein secondary structure. Therefore, conformational changes that occur upon ligand binding may be measured by CD [45]. If heme binding causes a significant conformational change, the binding affinity may be measured by varying the concentration of ligand or substrate [46]. Free heme is symmetric and does not produce a CD signal. However, heme may be held in an asymmetric environment in a protein binding pocket, causing it to produce a CD signal in its own absorbance range (~350–450 nm) [42]. The benefit of this method is that it detects reversible binding and it can be used to monitor binding over time. The drawback of CD is that if heme binding does not cause chiral changes, binding will not be detected.

### 2.8. Size Exlusion Chromatography

Size exclusion chromatography is another simple method to study heme binding. The method which yields the most information is high pressure liquid chromatography (HPLC). HPLC can detect formation of heme-protein complexes, help determine binding stoichiometry, and detect protein complex formation in response to ligand binding. While informative, HPLC does not provide enough data to calculate K_d_ in a single run; however, it is a very useful tool to qualitatively detect heme binding. In HPLC, a sample of protein and heme is passed through a size exclusion column and the eluate is monitored for protein and heme. Most proteins can be detected by absorbance at 280 nm, and heme can be detected by its absorbance in the region of 400 nm. Co-elution of heme and protein suggests heme binding [11,27], and integration of the elution peaks (or concentration measurements of the sample fractions) can elucidate the binding stoichiometry [32]. A second benefit of HPLC is that it can detect heme-dependent formation of protein dimers and complexes [39]. This is not an equilibrium method and it may be expected that very weak, dynamic binding sites may lose their heme during the few hours it takes to run through a typical column. However, this method has been used to successfully detect reversible heme binding [27]. The experiment could be performed at equilibrium by including heme in the column buffer so the protein sample will experience a constant concentration of heme as it travels through the column. In this manner, a heme binding protein would produce a surge in heme elution concurrent with protein. This peak can be integrated to determine how much heme bound to the protein at equilibrium. This particular technique does not appear to be favored, likely due to the “sticky” nature of heme which may persist in the HPLC machinery and column if used in the running buffer itself. A wash with basic buffer may help if this is a problem. In addition, if binding is weak, it may still be difficult to detect using this equilibrium method because of the high baseline of heme present throughout the experiment.

Buffer exchange columns provide a much simpler, faster assay for heme binding, although they do not yield retention times (loosely tied to molecular weight) as HPLC does. This method uses buffer exchange columns (e.g., PD-10 columns) designed to quickly elute large molecules (such as proteins) while retaining common buffer molecules (including heme). This allows for the purification of heme-protein complexes from a mixture of protein and heme. The run time is typically 2 min by centrifugation, and the amount of protein and heme in the eluate can be measured afterward [32,37].

Heme-agarose beads provide a useful assay to detect heme binding proteins by affinity purification. After the sample is incubated with the beads, the supernatant (containing components which did not bind to the beads) may be collected or discarded, then the beads are gently washed. Any proteins bound to the heme beads are then removed by denaturing with heat and SDS gel loading buffer. The heme binding proteins are detected by sodium dodecyl sulfate polyacrylamide gel electrophoresis (SDS PAGE) [27,37,46,47,48].

### 2.9. Isothermal Titration Calorimetry

Isothermal titration calorimetry (ITC) is a powerful technique often used in ligand binding studies. ITC provides thermodynamic data (temperature change due to binding) used to calculate the changes of enthalpy, entropy, and free energy of binding, as well as K_d_ and stoichiometry. The experimental considerations of ITC are similar to those of spectroscopic studies, with the significant exception of buffer composition. In ITC, it is important that the titrant and sample buffer components and concentrations are the same because any differences will produce a signal as the two are mixed. For this reason, protein samples are often buffer exchanged into the same buffer as the heme [29,35,49,50,51,52].

### 2.10. Advanced Methods

So far, the methods discussed are suitable as assays for heme binding. These methods are relatively simple and should yield results regardless of the specifics of the regulatory mechanism (with the exception of circular dichroism). Below is a summary of techniques which can be even more informative, but are more complex or are dependent on the regulatory mechanism.

Analytical ultracentrifugation and gel mobility shift assays can clearly show the effects of heme on protein complexes. Such changes will also be visible in size exclusion chromatography as mentioned above. Since these methods detect changes in protein complexes, they are not susceptible to non-specific binding and are therefore more convincing as positive indicators of heme binding and regulation [19,30,32,36,39,53].

More advanced spectroscopic methods can provide a wealth of chemical and structural information about heme binding. Magnetic circular dichroism, electron paramagnetic resonance, and Raman spectroscopy are used to study bound heme to determine the iron ion’s oxidation state, spin, coordination geometry, and axial ligands (e.g., protein residues, water, CO, NO, etc.) [9,30,32,33,34,39,41,54,55]. X-ray crystallography and multidimensional NMR can provide detailed structures and elucidate conformational changes [29,36,41,56,57]. Proteins can have different affinities for reduced versus oxidized heme, leading to different regulatory responses [54]. Thus, knowing the oxidation state of bound heme may be crucial to understanding a given pathway. Detailed structural information can provide a clear picture of heme regulation mechanics and assist in the development of new drugs.

### 2.11. Monitoring Heme In-Vivo

In recent years, heme-sensitive fluorescent reporters have been designed to detect free or “labile” heme pools within living cells [58]. The simplest method was a heme-quenched, genetically encoded sensor consisting of a fusion of Enhanced Green Fluorescent Protein (EGFP) and cytochrome b562 [44]. This sensor was expressed in *S. cerevisiae* and used cytochrome b562 to bind heme, which subsequently quenched the nearby EGFP. This method was proven to be useful, but suffered from poor signal due to the lack of an internal control, difficulty establishing saturation (because the signal decreases with increasing heme), and sensitivity to heme-related metabolites at high concentrations [44,58].

A similar system was developed and improved by the addition of a heme-insensitive fluorescent domain (mKATE2) to the EGFP-cytochrome b562 fusion. This addition acts as an internal control [59]. In addition, the cytochrome sequence was mutated to produce a sensor with a K_d_ in the nM range, typical of heme-regulated proteins and regulatory heme pools [60]. The system was also expanded by adding targeting sequences, allowing imaging of heme concentrations in the cytosol, nucleus, and mitochondria. Hanna et al. also point out that while current heme sensors may have different affinities for ferric vs. ferrous heme, they still bind both. There is a need for sensors specific to ferric or ferrous heme.

Another group has developed a sensor which results in a positive fluorescence signal upon specific heme binding. Rather than using a single heme binding protein as the sensory domain, Song et al. used two proteins which interact to transfer heme across the membrane of *B. anthracis*. During heme uptake by *B. anthracis*, heme is transferred between IsdX1 and IsdC via their respective NEA iron Transporter (NEAT) domains. During the exchange, a transient IsdX1:heme:IsdC complex is formed. In order to use this interaction as an in vivo heme sensor, Song et al. created a fusion protein expressed in HeLa cells consisting of the NEAT domains of IsdX1 and IsdC placed between two fluorescent proteins, ECFP and EYFP (Enhanced Cyan/Yellow Fluorescent Protein). Upon heme binding, the two NEAT domains form a heterodimer, bringing the two fluorescent domains into close proximity. This allows for energy transfer from the excited ECFP domain to the EYFP domain. The ratio of cyan and yellow fluorescence provides the output signal. The K_d_ of this fluorescent heme probe is in the nanomolar range, suitable for detecting regulatory heme binding. Finally, the probe was successfully targeted to the mitochondrial matrix, endoplasmic reticulum, and the nucleus [61].

Another probe was developed in the malarial parasite *Plasmodium falciparum*, in which the sensor domain was based on a sequence from the parasite’s heme binding protein Histidine Rich Protein 2 (PfHRP2) [62]. Similar to the use of cytochrome b562 by Hanna et al., the PfHRP2 heme sensor was placed between ECFP and EYFP. This method also resulted in fluorescence quenching which was proportional to heme concentration. Upon further optimization, this probe provided new insights into the tight regulation of heme concentration in the parasite and the mechanics of antimalarial drugs [62]. Together, the imaging methods discussed above are very promising. The ability to understand how various mutations, cell cycles, environmental changes, and developmental stages affect heme concentrations will provide a bigger picture of heme regulation.

## 3. Conclusions

Heme is an important signaling molecule which regulates many cellular pathways. Our understanding of cellular responses to oxidative stress, energy demands, metabolic stress, and hypoxia continues to grow as we learn how heme regulates relevant pathways. It can be difficult to determine where in a pathway heme regulation occurs directly, but many common methods have been tailored to detect reversible heme-protein binding. Because of the small binding constants of regulatory heme binding, and the “sticky” nature of heme, it is important to plan and interpret these experiments carefully and to confirm them in vivo. Heme binding to a particular protein can then be studied further to understand the biochemical mechanisms involved in regulation. In addition, live cell imaging of heme allows us to study regulatory heme pools at the cellular and subcellular level. Such fluorescent imaging techniques may prove extremely useful for understanding events and diseases which cause changes in localized heme concentrations. Together, these tools are allowing us to understand the vital part that heme plays in cellular signaling. Our hope is that a better understanding of heme regulation will speed the development of therapies and treatments for cancers and other diseases.
